# Supplementary material for: The effects of spatial and temporal replicate sampling on eDNA metabarcoding
Source: PeerJ. 2019 Jul 26;7:e7335. doi: 10.7717/peerj.7335 (PMC6662575; doi:10.7717/peerj.7335)
Supplement: Supplemental Information 1 — Sequences for primers used in the first round amplification. First round primers were modified to include additional bases between template-specific primer and the Illumina tail, to allow for demultiplexing on primer sequence during data processing (highlighted in bold). [file peerj-07-7335-s001.docx]

| First Round | | |
| --- | --- | --- |
| Primer | **Direction** | **Sequence** (Universal tail – [modification] – template-specific primer) |
| BF1-ill1 | Forward | TCGTCGGCAGCGTCAGATGTGTATAAGAGACAG **[ATGG]** ACWGGWTGRACWGTNTAYCC |
| BF1-ill2 | Forward | TCGTCGGCAGCGTCAGATGTGTATAAGAGACAG **[CGT]** ACWGGWTGRACWGTNTAYCC |
| BF1-ill3 | Forward | TCGTCGGCAGCGTCAGATGTGTATAAGAGACAG **[TC]** ACWGGWTGRACWGTNTAYCC |
| BF1-ill4 | Forward | TCGTCGGCAGCGTCAGATGTGTATAAGAGACAG **[G]** ACWGGWTGRACWGTNTAYCC |
| BR2-ill1 | Reverse | GTCTCGTGGGCTCGGAGATGTGTATAAGAGACAG **[ATGGA]** TCDGGRTGNCCRAARAAYCA |
| BR2-ill2 | Reverse | GTCTCGTGGGCTCGGAGATGTGTATAAGAGACAG **[CGA]** TCDGGRTGNCCRAARAAYCA |
| BR2-ill3 | Reverse | GTCTCGTGGGCTCGGAGATGTGTATAAGAGACAG **[TC]** TCDGGRTGNCCRAARAAYCA |
| BR2-ill4 | Reverse | GTCTCGTGGGCTCGGAGATGTGTATAAGAGACAG **[G]** TCDGGRTGNCCRAARAAYCA |
| Second Round | | |
| Primer | **Direction** | **Sequence** (Illumina adapter – index – universal tail) |
| NEX-F | Forward | AATGATACGGCGACCACCGAGATCTACAC **[i5 index]** TCGTCGGCAGCGTC |
| NEX-R | Reverse | CAAGCAGAAGACGGCATACGAGAT **[i7 index]** GTCTCGTGGGCTCGG |
