# Supplementary material for: The effects of spatial and temporal replicate sampling on eDNA metabarcoding
Source: PeerJ. 2019 Jul 26;7:e7335. doi: 10.7717/peerj.7335 (PMC6662575; doi:10.7717/peerj.7335)
Supplement: Supplemental Information 2 [file peerj-07-7335-s002.docx]

| Samples | First round | Second round |
| --- | --- | --- |
| Week 1-5 (2 May to 30 May) | BF1-ill1 / BR2-ill1 | Nextera XT, set C (N701-715 / S513-522) |
| Week 6-10 (6 June to 4 July) | BF1-ill2 / BR2-ill2 | Nextera XT, set C (N701-715 / S513-522) |
| Week 11-15 (11 July to 8 August) | BF1-ill3 / BR2-ill3 | Nextera XT, set C (N701-715 / S513-522) |
| Week 16-20 (15 August to 12 September) | BF1-ill4 / BR2-ill4 | Nextera XT, set C (N701-715 / S513-522) |
